# HO-1 reduces heat stress-induced apoptosis in bovine granulosa cells by suppressing oxidative stress

**DOI:** 10.18632/aging.102136

**Published:** 2019-08-12

**Authors:** Yiru Wang, Caixia Yang, Nahla Abdalla Hassan, Elsheikh, Chengmin Li, Fangxiao Yang, Genlin Wang, Lian Li

**Affiliations:** 1 College of Animal Science and Technology, Nanjing Agricultural University, Nanjing 210095, China

**Keywords:** HO-1, heat stress, reactive oxygen species (ROS), granulosa cells, apoptosis

## Abstract

Heat stress negatively affects reproduction in cattle by disrupting the normal function of ovarian granulosa cells (GCs), ultimately leading to oxidative damage and cell death via apoptosis. Heme oxygenase-1(HO-1) is a member of the heat shock protein family, which are associated with cellular antioxidant defenses and anti-apoptotic functions. Recent studies demonstrated that HO-1 is upregulated in heat-stressed cells. In the present study, we investigated the expression of HO-1 in bovine GCs transiently exposed to heat stress and characterized the expression and activity of key oxidative stress enzymes and molecules. We show that heat stress induced oxidative stress and apoptosis, and enhanced Nrf2 and HO-1 expression in primary GC cultures. Knocking down HO-1 expression using siRNA exacerbated both oxidative stress and apoptosis, whereas pre-treating GCs with hemin, which induces HO-1 expression, partially prevented these effects. These findings demonstrate that HO-1 attenuates heat stress-induced apoptosis in bovine GCs by decreasing production of reactive oxygen species and activating the antioxidant response.

## INTRODUCTION

High ambient temperature is considered to be a critical factor contributing to reduced fertility in cattle in tropical and subtropical countries, although the involvement of heat stress in this phenomenon is well documented even in regions with temperate climates [1–3]. Heat stress influences ovarian function, estrous expression, oocyte health, and embryonic development [4, 5]. Mammalian ovarian follicles are surrounded by granulosa cells (GCs) and theca cells, which produce signals and hormones that enable oocyte competency to develop into the blastocyst stage [6, 7]. Normal proliferation and differentiation of GCs are crucial for optimal follicular growth, oocyte development, ovulation, and luteinization [8, 9]. Several studies reported that heat stress adversely affects ovarian GCs, by inducing oxidative damage, endoplasmic reticulum stress, and apoptosis [10–12].

Oxidative stress results from imbalances between the generation and elimination of reactive oxygen species (ROS) within cells; excessive ROS generation can overload cellular antioxidant defenses and damage lipids, proteins and DNA, thereby disrupting normal cell function and causing cell death via apoptosis or necrosis [13]. ROS generation and oxidative stress are critically involved in heat stress-induced apoptosis [14, 15]. Heme oxygenase 1 (HO-1), also known as heat shock protein-32 (Hsp32), is a stress-inducible enzyme that plays important roles in iron homeostasis, antioxidant defense, and apoptosis prevention [16–18]. Research has shown that HO-1 acts as an antioxidant in hepatocytes [19] and that low serum HO-1 levels are associated with an increased risk for polycystic ovarian syndrome [20]. Recent studies have addressed the molecular mechanisms responsible for the cytoprotective effects of HO-1 against apoptosis, and have suggested its potential relevance as a drug target in anti-oxidative therapies [21, 22].

Our previous studies indicated that heat stress can induce apoptosis of ovarian GCs and activate the expression of HO-1, but the precise molecular mechanism involved remained unclear [10, 12]. In the present study, we hypothesized that HO-1 expression mediates anti-apoptotic effects in heat-treated GCs by decreasing oxidative stress. Using a bovine GC culture system, we confirmed the impact of heat stress on apoptosis and characterized the cytoprotective mechanism of HO-1 in relation to the expression and activity of key oxidative stress enzymes and markers, including SOD, GSH-Px, and MDA.

## RESULTS

### Heat stress induces ROS generation and apoptosis in GCs

**Figure 1:**
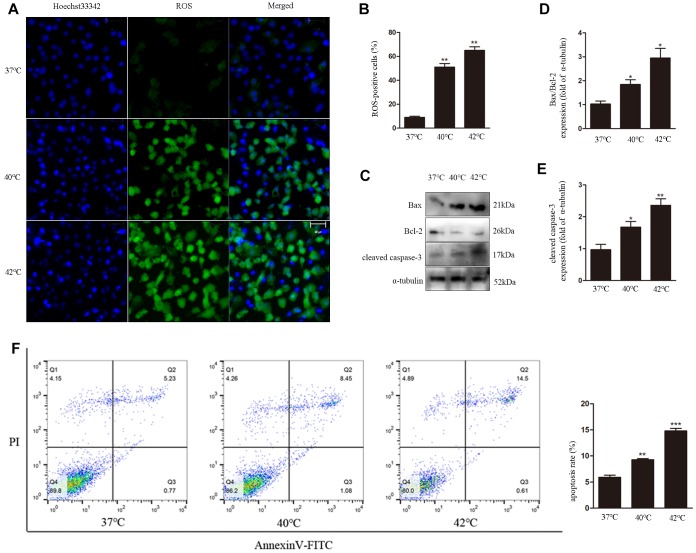
**Heat stress induces ROS generation and apoptosis in bovine ovarian GCs.** (**A**, **B**) Intracellular accumulation of ROS at different temperatures, quantified by DCF fluorescence. Scale bars, 50 μm. (**C**–**E**) Immunoblot analysis of Bax/Bcl-2 and cleaved caspase-3in GCs. (**F**) Apoptosis induction by heat stress in GCs, analyzed by FACS assay. Data represent mean ± SEM; n = 3 in each group. *P < 0.05; **P < 0.01; ***P < 0.001.

To investigate the effects of heat stress on ROS generation by GCs, DCF fluorescence was evaluated as a surrogate measure of ROS production in cultured bovine GCs. We found that ROS generation increased with increasing temperatures, i.e. 37°C < 40°C < 42°C (Figure 1A, 1B). In parallel, western blot analyses revealed apoptosis induction, denoted by upregulation of cleaved caspase-3 and increased Bax/Bcl-2 ratio (Figure 1C–1E). Furthermore, the increase in the apoptotic rate with increasing temperatures was also confirmed through Annexin V/PI staining using flow cytometry (Figure 1F).

### Heat stress induces alterations in cellular redox status and promotes Nrf2 nuclear translocation in GCs

**Figure 2:**
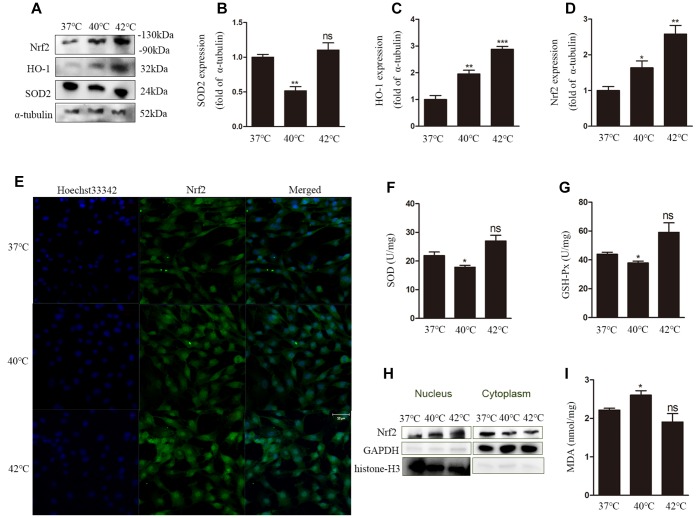
**Heat stress leads to dysfunction of the antioxidant defense system and oxidative stress in GCs.** (**A**–**D**) Western blotting showing the expression of Nrf2, HO-1, and SOD2. (**E**) Nuclear translocation of Nrf2 evaluated by DCF fluorescence. Scale bars, 50 μm. (**F**) Estimation of SOD activity. (**G**) Estimation of GSH-Px activity. (**H**) Nuclear translocation of Nrf2 determined by western blotting. (**I**) MDA content. Data represent mean ± SEM; n = 3 in each group. *P < 0.05; **P < 0.01; ***P < 0.001; ns, not significant.

The redox status in cells is determined by the balance between oxidant stressors and antioxidant reserves. We investigated MDA levels, an indicator of oxidant stress, as well as SOD and GSH-Px activities as a measure of antioxidant cellular reserves. To confirm the effect of heat stress on the antioxidant defense system**,** the expression of the antioxidant gene SOD2 was also analyzed in GCs by western blot. Compared to control cells (37°C), SOD2 expression was significantly decreased (Figure 2A, 2B), and SOD activity was reduced (Figure 2F) in GCs exposed to heat stress (40°C). In addition, heat stress (40°C) resulted in markedly lower GSH-Px activity and higher MDA levels than those measured in the normothermic control group (Figure 2G, 2I). In contrast, compared to control cells, at 42°C the levels of SOD and GSH-Px were increased, while those of MDA were decreased, although these changes were not statistically significant.

Next, the expression of nuclear factor erythroid 2-related factor 2 (Nrf2) and HO-1was measured at the protein level. The expression of both proteins was increased significantly after heat stress (Figure 2A, 2C, 2D), a condition that also promoted nuclear translocation of Nrf2 (Figure 2E, 2H).

### HO-1knockdown enhances heat stress-induced ROS generation and apoptosis, and reduces the antioxidative response

**Figure 3:**
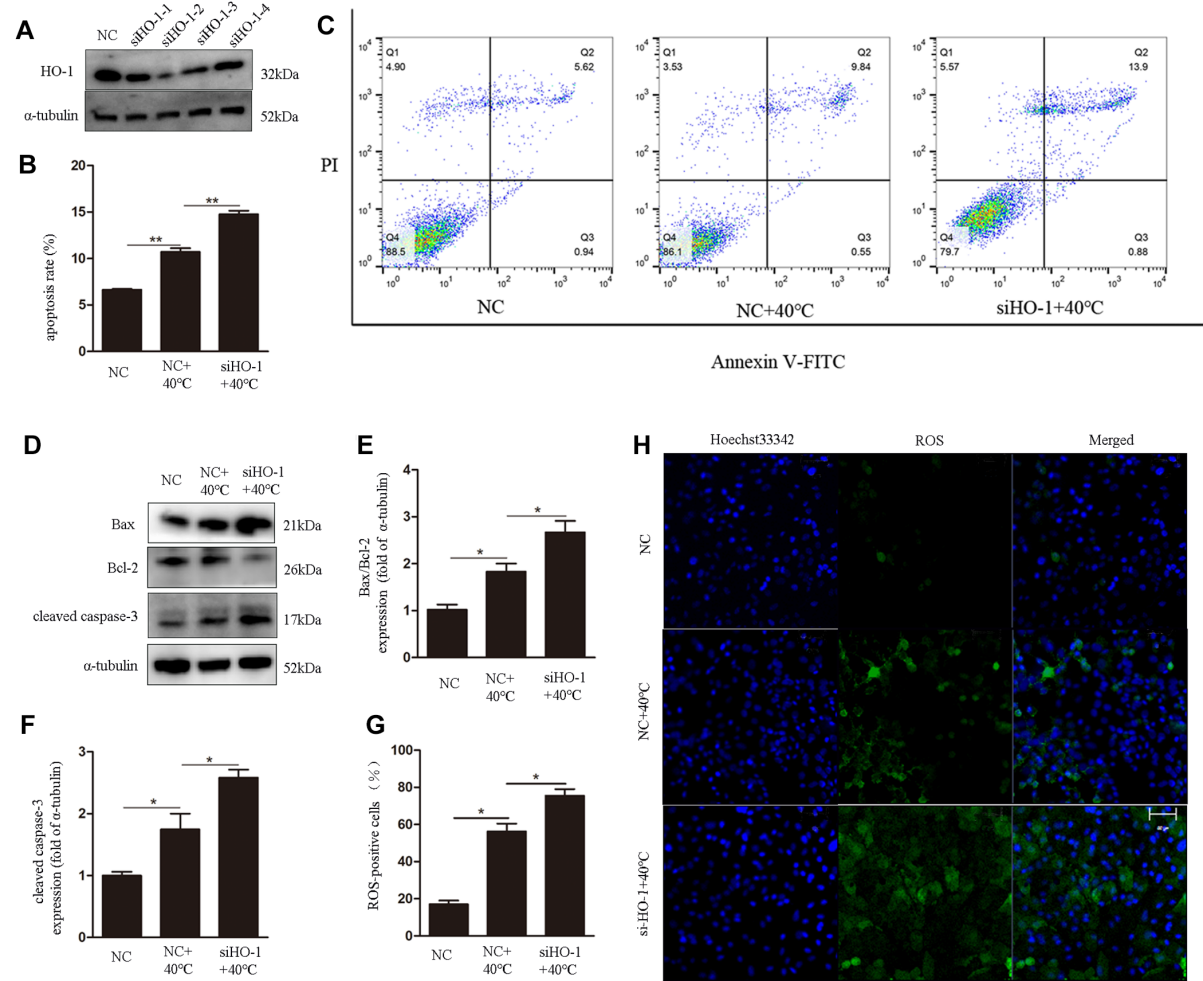
**HO-1 gene knockdown enhances ROS generation and induces apoptosis in GCs under heat stress.** (**A**) Western blot analysis of HO-1 expression after siRNA mediated knockdown of HO-1. (**B**, **C**) Annexin V/PI FACS analysis of apoptosis. (**D**–**F**) Expression of Bax/Bcl-2 and cleaved caspase-3 by western blot. (**G**, **H**) Intracellular ROS accumulation detected through DCF fluorescence. Scale bars, 50 μm. Data represent mean ± SEM; n = 3 in each group. *P < 0.05; **P < 0.01; ***P < 0.001.

**Figure 4:**
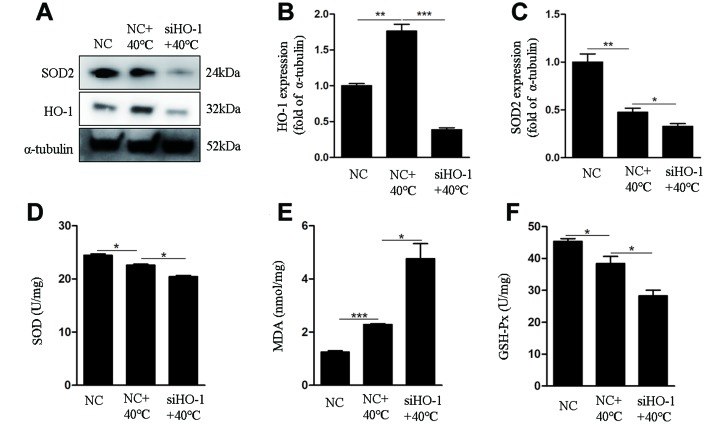
**HO-1 gene knockdown impairs antioxidant defenses in GCs exposed to heat stress.** GCs were transfected with NC or siHO-1 and exposed to heat stress for 2 h. (**A**–**C**) Western blot expression of HO-1 and SOD2. (**D**) SOD activity. (**E**) MDA content. (**F**) GSH-Px activity. Data represent mean ± SEM; n = 3 in each group. *P < 0.05; **P < 0.01; ***P < 0.001.

To assess the role of HO-1 on oxidative stress responses and apoptosis under heat stress, its expression was silenced using specific siRNAs. We used siHO-1-2 in these experiment because it had the strongest knockdown effect (Figure 3A). Annexin V/PI staining showed that the apoptotic rate in the siHO-1 + 40°C group of cells was increased significantly compared to the NC (negative control siRNA) + 40°C group (Figure 3B, 3C). Concomitantly, and compared to the latter control, significant increases in the expression of cleaved caspase-3 and the Bax/Bcl-2 ratio (Figure 3D–3F), as well as enhanced ROS production (Figure 3G, 3H), were observed in siHO-1 + 40°C cells. To investigate whether HO-1 silencing affects the redox status of GCs, SOD2 expression, MDA content, and SOD and GSH-Px activities were next measured. Western blot confirmed effective silencing of HO-1 expression in siHO-1-transfected GCs, and a significant decrease in SOD2 expression in the siHO-1 + 40°C group of cells (Figure 4A, 4C). This result was consistent with reduced SOD levels in HO-1-silenced, heat stress GCs (Figure 4D). In addition, compared to the NC + 40°C group, MDA levels were significantly up-regulated (Figure 4E) and the activity of GSH-Px was significantly down-regulated (Figure 4F) in the siHO-1 + 40°C group.

### HO-1induction increases antioxidant defenses and attenuates ROS generation and apoptosis in heat-stressed GCs

**Figure 5:**
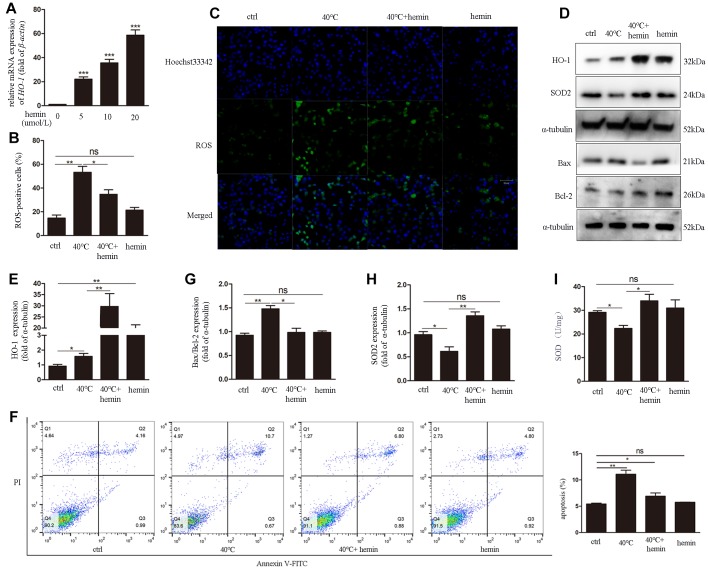
**Hemin-mediated HO-1 induction decreases oxidative stress and attenuates ROS generation and apoptosis in GCs exposed to heat stress.** (**A**) qRT-PCR analysis of *HO-1* gene expression in GCs pre-treated with hemin (10 μmol/L for 48h). (**B**, **C**) Effect of HO-1 overexpression on ROS accumulation in GCs under heat stress. (**D**, **E**, **G**, **H**) HO-1, SOD2, and Bax/Bcl-2 expression determined by western blotting. (**F**) Hemin pre-treatment reduced apoptosis of heat-stressed GCs, as determined by FACS assay. (**I**) Enhanced SOD activity in hemin-treated GCs. Data represent mean ± SEM; n = 3 in each group. *P < 0.05; **P < 0.01; ***P < 0.001; ns, not significant.

To confirm the protective effects of HO-1 against oxidative stress and apoptosis triggered by heat stress, hemin (an HO-1-specific activator) was applied to cultures over 48 h to induce HO-1 expression in GCs (Figure 5A). After heat stress treatment (40°C), significant decreases in both ROS generation (Figure 5B, 5C) and apoptotic rate (Figure 5F) were detected in cells treated with hemin. Meanwhile, western blots confirmed HO-1 induction and decreased Bax/Bcl-2 ratio in the 40°C + hemin group (Figure 5D, 5E, 5G). In addition, compared to the 40°C control group, the expression of SOD2 was dramatically up-regulated (Figure 5D, 5H) and SOD levels were increased (Figure 5I) in GCs in the 40°C + hemin group.

## DISCUSSION

Despite the use of modern cooling systems in dairy farms, heat stress remains a major contributing factor to the lowfertility among lactating dairy cows in hot environments [23–25]. Noxious effects of heat stress include impaired steroidogenic ability [26], altered follicular dynamics that impact GC function [10], and deficient oocyte maturation, fertilization, and preimplantation embryonic development [27, 28]. In the ovarian follicle, GCs play a vital role in nourishing the oocytes and secreting estrogens to establish a suitable microenvironment for normal reproductive function [8, 9]. GC apoptosis due to heat stress may be one of the most critical factors affecting GC function and dairy cow fertility [11].

The present study sheds light on the deleterious effects of heat stress on GC function and survival, revealing a protective role for HO-1 against oxidative damage and apoptosis. Previous research demonstrated that oxidative stress plays a pivotal role in heat stress-induced apoptosis [29, 30], and the involvement of mitochondrial pathways have been reported in mouse GCs exposed to high temperatures in vitro [12]. In the present study, GCs weresubjected to simulated heat stress(either 40°C or 42°C), which led to oxidative stress and apoptosis. Oxidative stress occurs when the steady-state ROS concentration is transiently or chronically enhanced, disturbing cellular metabolism and its regulation and damaging cellular constituents [13]. Accordingly, heat-stressed GCs showed increased ROS production, decreased SOD2 expression, and reduced activities of SOD and GSH-Px, two key enzymes in the cellular antioxidant system. Mitochondrial dysfunction was involved in the ensuing apoptosis of GCs, as indicated by increases in both cleaved caspase-3 expression and the Bax/Bcl-2 ratio [12–14].

Interestingly, the expression of two oxidative biomarkers, Nrf2 and HO-1, was found to be upregulated in GCs exposed to heat stress. This is in line with results of an earlier study, which showed that hypoxia induces significant upregulation of Nrf2-mediated oxidative stress response genes in the bovine embryo [32]. Furthermore, our study showed that heat stress promotes Nrf2 nuclear translocation. Nrf2 controls the transcription of the HO-1 gene [33], which encodes a cytoprotective heat shock protein (HSP) found to be upregulated by oxidative stress and inflammation [34]. The present data also confirmed that HO-1 expression can be induced by heat stress. GCs exposed to 40°C exhibited significantly higher MDA content and lower SOD and GSH-Px activities than control cells cultured in normothermic conditions. This is consistent with oxidative stress-mediated inhibition of antioxidant genes and production of MDA, a by-product of lipid peroxidation. However, in cells grown at 42°C, neither MDA content nor SOD and GSH-Px activities differed significantly from control, this may be due to that these antioxidant enzymes were further upregulated to neutralize excessive ROS formation [36]. However, since both ROS generation and apoptosis were enhanced at 42 relative to control, the compensatory upregulation of SOD and GSH-Px activities at higher temperatures was clearly unable to counteract cell death.

**Figure 6:**
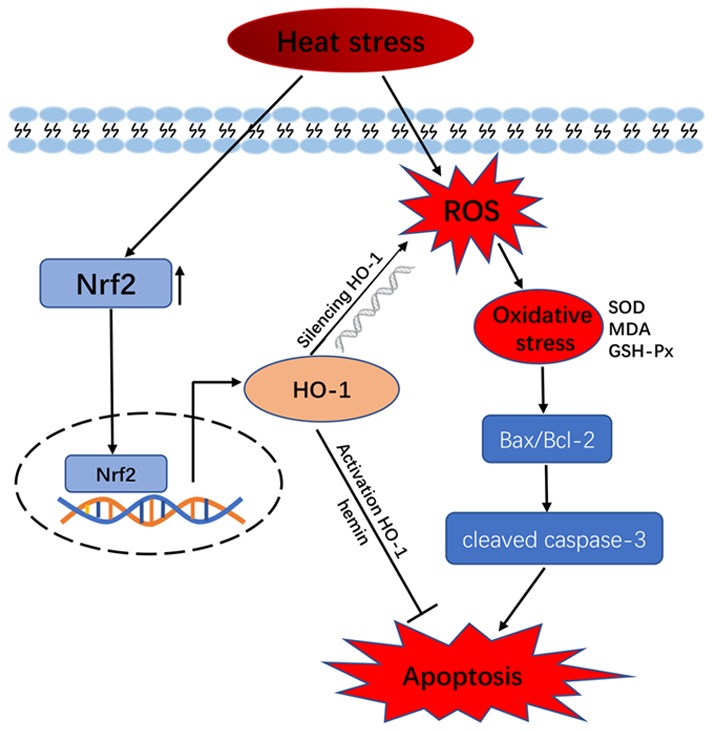
**Schematic model of HO-1 regulation of oxidative stress and apoptosis in GCs exposed to heat stress.**

Our previous study showed that heat stress increases the synthesis of several HSPs; i.e., HSP32 (HO-1), HSP60, HSP70, HSP90, and HSP105, which help maintain cellular redox homeostasis to ensure survival of cells [34]. HO-1 is an oxidative stress marker and contributes to iron homeostasis, antioxidant defense, and apoptosis prevention. Unlike HO-2, HO-1 is inducible and was suggested to be an important autocrine/paracrine factors that regulates apoptosis in porcine GCs [33]. Despite evidence for a role of HO-1 in the induction of genes involved in oxidative stress response pathways triggered by ROS accumulation in cells [10, 31], its role in heat stress in GCs had not been investigated. Interestingly, we show here that HO-1 knockdown potentiates heat stress-mediated oxidative stress and apoptosis in GCs, while pre-treatment with hemin (a HO-1 inducer) had protective effects. These results imply that HO-1 counteracts heat stress-induced apoptosis by decreasing oxidative stress (Figure 6).

In summary, we found that heat stress induces apoptosis in bovine GCs by increasing intracellular ROS and ROS and decreasing the expression and activity of antioxidant enzymes. Under heat stress, HO-1 silencing enhances ROS production and apoptosis, while forced expression decreases apoptosis by attenuating ROS accumulation and upregulating the expression/activity of antioxidant enzymes. Thus, modulation of HO-1 expression may be a promising approach to the prevention and treatment of ovarian dysfunction and infertility caused by heat stress in cow.

## METHODS

### GC isolation, culture, and treatments

This study was approved by the Animal Protection and Utilization Committee of Nanjing Agricultural University, Nanjing, China. All ovaries were acquired from Holstein cattle. After acquisition, the ovaries were washed with saline and the follicular fluid was extracted from follicles (about 5 mm in diameter) and placed into a 15 mL centrifuge tube using a disposable needle. Follicles were healthy and round with a sharp and continuous granulosa cell membrane [37], indicating that they were well developed. Follicular fluids were clear. Follicular cells were washed twice with phosphate buffered saline (PBS) and resuspended in culture medium (DMEM-F12, HyClone, Logan, USA). Cells were then plated into T25 flasks (5 × 10^6^ cells/flask) in DMEM-F12 supplemented with 10 % fetal bovine serum (FBS, Gibco, Gaithersburg, USA) and cultured at 37°C with 5 % CO_2_. The medium was replaced after 24 h in order to remove non-adherent cells. The cells remaining were approximately triangular or polygonal, with large nuclei, and expressed follicle stimulating hormone receptor (FSHR), a specific marker of GCs (Supplemental Figure S1). After two or three days, cells at passage II were used for experiments (within one week). In order to establish a heat stress model, GCs were heat-treated (40°C and 42°C) for 2 h and then allowed to recover for 6 h at 37°C [12, 38]. For pharmacological experiments, before heat treatment GCs were pre-treated with 10 μmol/L hemin (Sigma, MO) for 48 h to induce HO-1 expression [39].

### Immunofluorescence staining

Cells were plated on coverslips, fixed with 4 % paraformaldehyde for 1 h and washed with PBS three times. After permeabilization with 0.5 % Triton X-100 for 20 min, cells were treated with 2 % bovine serum albumin (BSA) in PBS for 1 h and incubated with anti-FSHR antibody (1:100; Proteintech, Chicago, USA) diluted in 2 % BSA at 4°C overnight. Finally, the cells were washed with PBS and incubated with a FITC-conjugated secondary antibody for 1 h in the dark. After washing with PBS, cells were stained with Hoechst 33342, mounted, and viewed under a 710 META laser-scanning confocal microscopy (Zeiss, Oberkochen, Germany).

### siRNA transfection

Negative control siRNA (NC-siRNA) and siRNAs targeting bovine HO-1 (HO-1-siRNA / siHO-1) were generated by QuanYang (Shanghai, China). GCs were seeded into six-well plates and cultured for 24 h until 60% confluence. Cells were then transfected with either 50 nmol/L HO-1-targeting siRNA or NC-siRNA using Lipofectamine 2000 reagent (Invitrogen, Carlsbad, USA) according to the manufacturer’s instructions. Successful depletion of HO-1 expression was confirmed by western blot analyses. After subsequent treatments, cells were harvested and analyzed.

### ROS staining assay

ROS accumulation was measured using DCFH-DA (Sigma, MO, USA), which is oxidized to fluorescent DCF by intracellular ROS. After treatments, the cells were washed with PBS three times and 10 μmol/L DCFH-DA in non-phenol red medium was added to the wells. After incubation for 30 min in the dark, the cells were washed three times with PBS, stained with Hoechst 33342 for 10 min, and viewed under laser-scanning confocal microscopy.

### Oxidative stress assay

The Biochemical Analysis Kit (Jiancheng Biotechnology, Nanjing, China) was used to measure malondialdehyde (MDA) content and superoxide dismutase (SOD) and glutathione peroxidase (GSH-Px) activities in GCs according to protocol instructions. Briefly, MDA quantification (expressed as nmol/mg) was based on the reaction of MDA with thiobarbituric acid in acidic medium at 95°C, detected by absorbance at 532 nm. SOD activity (expressed as U/mg) was determined spectrophotometrically at 550 nm using the xanthine/xanthine oxidase system. GSH-Px activity (expressed as U/mg) was measured by quantifying the absorbance (412 nm) of the complex formed by the reaction between glutathione and 5, 5-dithiobis-(2-nitrobenzoic).

### Apoptosis assay

Flow cytometry was used to analyze apoptosis in GCs using an Annexin V/PI apoptosis detection kit (MACS, Miltenyi Biotec, Bergisch Gladbach, Germany) according to the manufacturer instructions. Briefly, GCs were seeded into bottles, treated, harvested, and washed once with PBS and twice with binding buffer. Cells were then incubated at room temperature with annexin V-FITC in the dark for 15 min and with PI for 1 min, and flow cytometry was performed immediately thereafter. The apoptotic rate is expressed as the sum of the percentage of early (Annexin V+/PI-) and late (Annexin V+/PI+) apoptosis cells.

### Quantitative RT-PCR (qRT-PCR)

Total RNA from GCs was extracted using TRIzol reagent (TaKaRa Biotechnology Co. Ltd., Tokyo, Japan) as described by the manufacturer. RNA concentrations were measured using a NanoDrop 2000 spectrophotometer (Thermo Scientific, Waltham, USA). Gene expression was measured by real-time PCR analysis using SYBR Premix Ex Taq (TaKaRa, DRR420A) on an ABI StepOne PCR system (Applied Biosystems, Foster City, USA). The relative expression of each target gene was normalized to that of *β-actin*. The primer sequences were as follows: *HO-1*forward primer 5′-CAAGGTGCAAGACTTGGCT-3′, reverse primer 5′-CCAGAAAGCTGAGTGTGAGG-3′; *β-actin* forward primer 5′-GAGGCTCAGAGCAAGAGAGG-3′, reverse primer 5′-TGCCAGATCTTCTCCATGTC-3′ (Supplementary Table S1).

### Western blot analysis

Cells were lysed on ice with RIPA Lysis Buffer (Beyotime, Nanjing, China) and supplemented with 1 % proteinase inhibitor (PMSF; Beyotime). Protein concentrations were determined using a BCA Protein Assay Kit (Beyotime). Samples containing 50 μg protein were separated on 6-12 % sodium dodecyl sulfate polyacrylamide gels (SDS-PAGE) and then electrotransferred onto polyvinylidene difluoride (PVDF) membrane (Millipore, Darmstadt, France). The membranes were blocked with 5 % non-fat milk in Tris-buffered saline + Tween 20 (TBST) for 1 h and incubated at 4°C overnight with primary antibodies: anti-HO-1 (1:500; Abcam, Cambridge, UK), anti-Bax (1:1000; Proteintech, Chicago, USA), anti-Bcl-2 (1:1000; Proteintech), anti- cleaved caspase-3 (1:500; Abcam), anti-SOD2 (1:1000; Cell Signaling Technology, Boston, USA), anti-Nrf2 (1:1000; Proteintech), anti-GAPDH (1:1000; Proteintech), anti-histone-H3 (1:2000; Proteintech), or anti-α-tubulin (1:2000; Cell Signaling Technology). The membranes were washed three times with TBS+ Tween 20 (TBST) for 10 min and incubated with a secondary horseradish peroxidase-conjugated antibody (1:2000, Proteintech) at 37°C for 1 h. Finally, the membranes were visualized using ECL Plus Reagent (Biosharp, Hefei, China) and the results quantified using an enhanced chemiluminescence detection system (Amersham, Piscataway, NJ). Proteins were quantified densitometrically with ImageJ software (National Institutes of Health, Bethesda, USA) and α-tubulin, GAPDH, and histone-H3 were used, as appropriate, as loading controls for normalization.

### Statistical analysis

Data are presented as the mean ± SEM unless indicated otherwise. Data were analyzed using a t-test and analysis of variance (ANOVA) with GraphPad Prism version 5.0 (GraphPad Software, San Diego, USA). P <0.05 was considered statistically significant.

## Supplementary Material

Supplementary Figure S1

Supplementary Table S1
