# Supplementary material for: HO-1 reduces heat stress-induced apoptosis in bovine granulosa cells by suppressing oxidative stress
Source: Aging (Albany NY). 2019 Aug 12;11(15):5535–47. doi: 10.18632/aging.102136 (PMC6710052; doi:10.18632/aging.102136)
Supplement: Supplementary Figure S1 [file aging-11-102136-s002.pdf]

SUPPLEMENTARY FIGURE

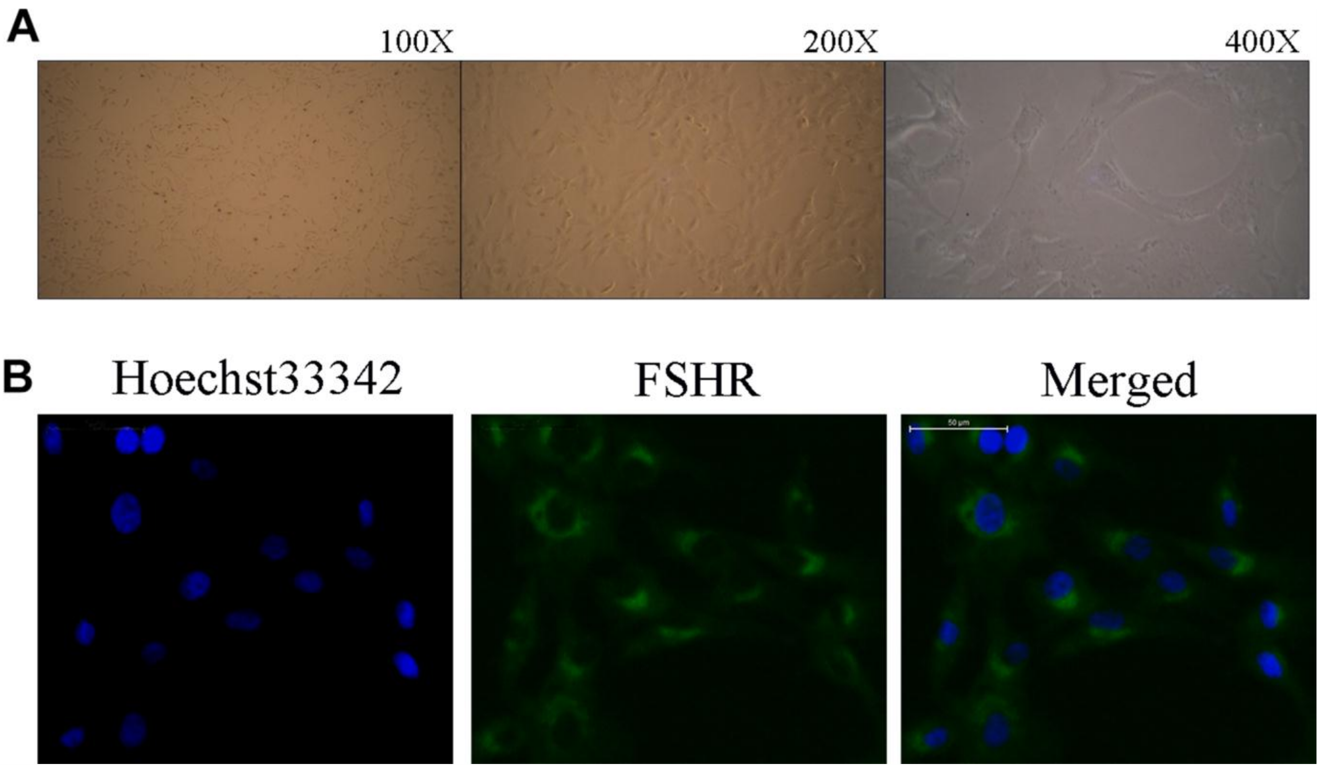

Supplementary Figure 1. Identification of ovarian granulosa cells (GCs) in dairy cows. (**A**) GCs visualized at different magnifications. (**B**) GCs specifically expressed FSHR.
