# Supplementary material for: HO-1 reduces heat stress-induced apoptosis in bovine granulosa cells by suppressing oxidative stress
Source: Aging (Albany NY). 2019 Aug 12;11(15):5535–47. doi: 10.18632/aging.102136 (PMC6710052; doi:10.18632/aging.102136)
Supplement: Supplementary Table S1 [file aging-11-102136-s001.pdf]

Supplementary Table S1. Primer sequences for HO-1.

Genes	Forward	Reverse
siHO-1-1	5'-GCUCAACAUCCAGCUGUUUTT-3'	5'-AAACAGCUGGAUGUUGAGCTT-3'
siHO-1-2	5'-CCAAGGACCAUGAUCCCUUTT-3'	5'-AAGGGAUCAUGGUCCUUGGTT-3'
siHO-1-3	5'-GCAGCAAGGUGCAAGACUUTT-3'	5'-AAGUCUUGCACCUUGCUGCTT-3'
siHO-1-4	5'-GGUCCUCACACUCAGCUUUTT-3'	5'-AAAGCUGAGUGUGAGGACCTT-3'
Negative control	5'-UUCUCCGAACGUGUCACGUTT-3'	5'- ACGUGACACGUUCGGAGAATT-3'
